# Lung Function and Respiratory Symptoms as Factors Associated with Smoking Cessation Status in a Population-Based Study

**DOI:** 10.3390/jcm15197502

**Published:** 2026-09-26

**Authors:** Denis Vinnikov, Irina Mukatova, Zhangir Dairuly, Aizhan Raushanova, Zhanna Romanova, Amil Suleimanov, Venerando Rapisarda, Zhanar Urazalina

**Affiliations:** 1 Environmental Health Laboratory, Al-Farabi Kazakh National University, 050040 Almaty, Kazakhstan; denisvinnikov@mail.ru (D.V.); d.zhangir@bk.ru (Z.D.); 2 Occupational Health Risks Laboratory, Peoples’ Friendship University of Russia (RUDN University), 117098 Moscow, Russia; 3 Department of Internal Diseases with Courses of Nephrology, Hematology, Allergology, and Immunology, Astana Medical University, 010000 Astana, Kazakhstan; mukatovair@mail.ru; 4 Department of Epidemiology, Biostatistics and Evidence-Based Medicine, Al-Farabi Kazakh National University, 050040 Almaty, Kazakhstan; aizhan.raushanova@kaznu.kz (A.R.); zhanna.romanova@kaznu.kz (Z.R.); amil.suleimanov90@gmail.com (A.S.); 5 Department of Public Health and Scientific Research, Khoja Akhmet Yassawi International Kazakh-Turkish University, 161200 Turkistan, Kazakhstan; 6 Department of Clinical and Experimental Medicine, Occupational Medicine, University of Catania, 95124 Catania, Italy; venerando.rapisarda@unict.it; 7 Department of Emergency Medicine, Semey Medical University, 071400 Semey, Kazakhstan

**Keywords:** smoking cessation, occupational, Kazakhstan, population-based

## Abstract

**Background/Objectives**: The aim of this study was to test the association of poorer spirometry and more respiratory symptoms with being an ex-smoker in a cohort of ever-smokers in a population-based study in Kazakhstan. **Methods**: We first enrolled 5058 subjects, of whom 1769 were classified as ever-smokers (63% current and 37% former smokers) from the general population in five cities in Kazakhstan and analyzed them hereinafter. They filled in a questionnaire and underwent spirometry with bronchodilation, in which z-scores of post-bronchodilation forced expiratory volume in one second (FEV_1_) and forced vital capacity (FVC) were analyzed. Respiratory symptoms and spirometry were compared between continuing smokers and those who stopped in multivariate logistic regression models. **Results**: Current smokers smoked a median of 10 (interquartile range (IQR) 7;20) cigarettes a day. In the fully adjusted models, neither lung function (FEV_1_, FVC and FEV_1_/FVC z-scores and even FEV_1_/FVC below the lower limit of normality (LLN)) nor symptoms could predict being an ex-smoker at present. Age (odds ratio (OR) 1.02; 95% confidence interval (CI) 1.01; 1.03), body mass index (BMI) over 25 kg/m^2^ (OR 1.36; 95% CI 1.10; 1.68), lifetime exposure to vapor, gas, dust and fume (OR 0.77; 95% CI 0.62; 0.97), exposure to SHS (OR 0.34; 95% CI 0.27; 0.42) and electronic cigarette use (OR 0.58; 95% CI 0.34;0.99) were significantly associated with being a former smoker vs. a current smoker at the time of examination. **Conclusions**: Spirometry and early respiratory symptoms did not predict being a former smoker, but co-exposure to secondhand smoke, occupational exposures, and electronic cigarette use sustain continuing smoking.

## 1. Introduction

Smoking cessation, both unaided and aided with either behavioral or pharmacological intervention, has been shown to be one of the most beneficial efforts in individual health improvement and primary prevention [1,2]. Quitting smoking increases life expectancy, improves quality of life and is highly beneficial for respiratory [3,4], cardiovascular [3] and other outcomes of an individual. Respiratory effects associated with smoking cessation, including lung function improvement [5,6,7] and symptom amelioration, are usually more pronounced in patients with existing chronic respiratory disease [8,9].

Medical doctors traditionally believe that advice to quit smoking supplemented with spirometry motivates patients to stop smoking soon after the intervention, and some studies confirm such an effect [10,11,12]. Critical analysis of the evidence, however, does not support the effect of spirometry-based communication on smoking harm [13,14]; nevertheless, spirometry and lung age communication may help identify early lung disease, where smoking is a modifiable risk factor. Given that chronic obstructive pulmonary disease (COPD) is a very prevalent disease [15], high-quality spirometry with some counseling on risk factors, including cigarette smoking, is believed to strengthen argumentation for a smoking COPD patient to stop smoking [16]. However, the effects of such interventions may be modified by peers’ attitudes, past attempts [17], occupational climate, state governmental policy and many other determinants. Therefore, spirometry alone or in combination with counseling may not yield the desired effect, but smokers with COPD will be more likely to cease smoking after the intervention compared to smokers with no COPD [18].

Respiratory symptoms, among other predictors, have been shown to increase the odds of successful quitting [19], more pronounced in COPD patients [20], but as with spirometry, studies are not consistent in such outcomes [21]. Because respiratory disease from smoking develops gradually, subjects exhibit some growing tolerance to cough, sputum and later even shortness of breath. Concurrently, growing tobacco addiction impedes successful smoking cessation attempts when a smoker complains of some symptoms. Therefore, both respiratory symptoms and spirometry may not fully explain the status of former smokers resulting from successful quit attempts in the past. In addition, some uncertainty remains whether symptoms and spirometry at very early stages urge subjects with no clinical diagnosis yet to contemplate quit attempts and even promote action.

We hypothesized that spirometric abnormalities and more advanced respiratory symptoms in subjects with no respiratory diagnosis at earlier stages may be associated with being an ex-smoker at the time of examination. In other words, we believe that ever-smokers with worse lung function and pronounced respiratory symptoms are more likely to be former smokers. This suggests that more of these individuals have tried to quit smoking during their lifetimes. Similarly, we expect that a group of subjects with better lung function and fewer symptoms would have fewer ex-smokers. Therefore, the aim of this study was to test the association of poorer spirometry and more respiratory symptoms with being an ex-smoker in a cohort of ever-smokers in a large population-based epidemiological study in Kazakhstan.

## 2. Materials and Methods

### 2.1. Study Design

The study was approved by the Committee on Bioethics of al-Farabi Kazakh National University (approval IRB-A387, dated 17 March 2022, extension granted 5 December 2024), and all participating subjects signed an informed consent form to participate. This was a population-based epidemiological study in five major parts of Kazakhstan, including the cities of Aktobe (west), Kostanay (north), Shymkent (south), Ust-Kamenogorsk (east) and Karagandy (center). In Kazakhstan, these five major locations differ considerably in geography, climate, population, income, economic structure and even the percentage of exposed population. We trained one medical doctor in each participating city in January 2025 to interview participants and then perform office spirometry using a standard protocol [22]. Subjects were invited by the outpatient doctors of the central outpatient facility by phone, whereas subjects were chosen using a computer randomizer from the enlisted population, usually 10,000 to 20,000 people residing in proximity to the facility. The initial list of residents included subjects aged 18 years and older.

**Figure 1:**
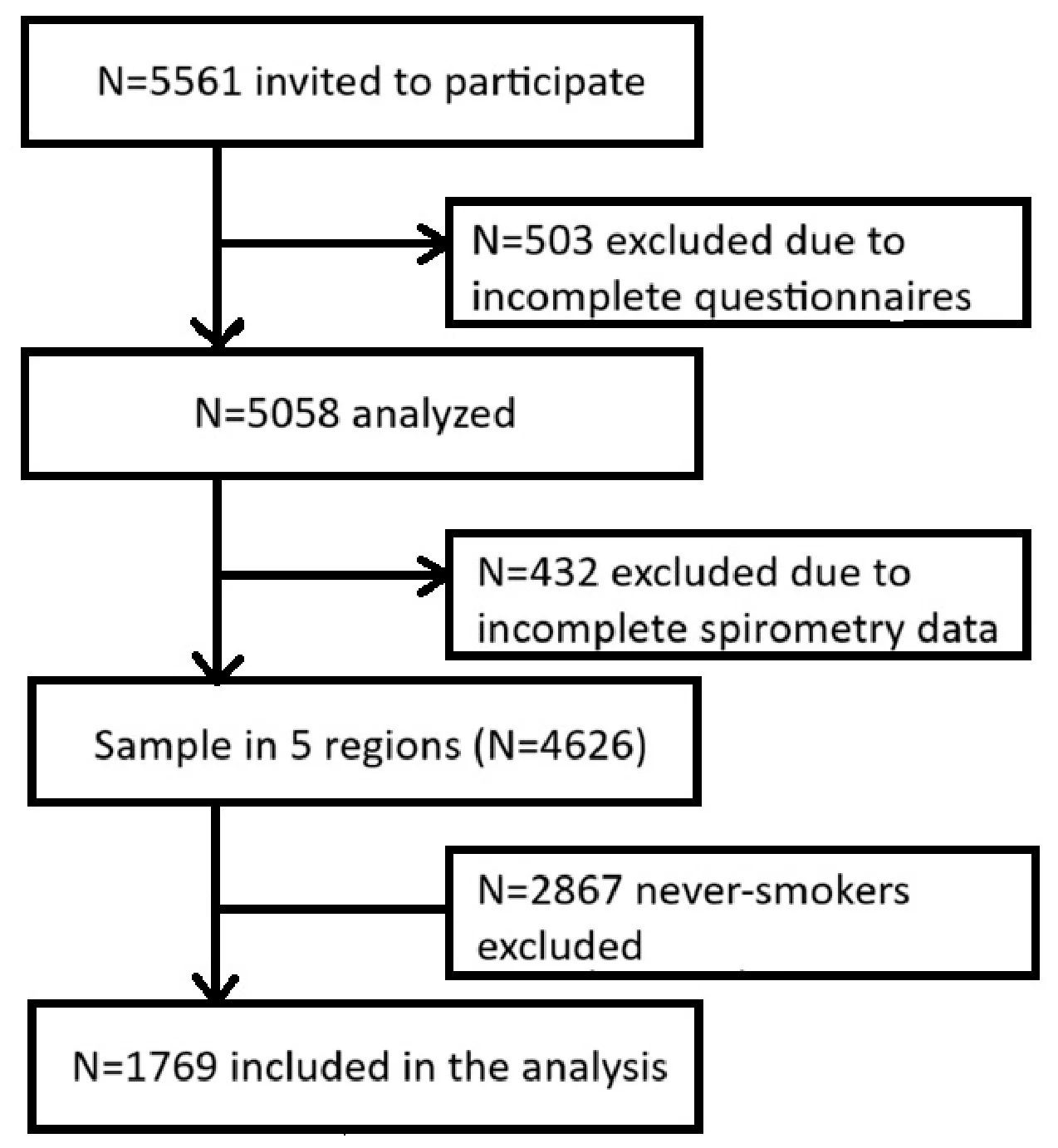
Flow diagram of participants’ recruitment and inclusion.

Overall, 5561 subjects were invited, seen by doctors and underwent spirometry in this study (Figure 1), and the sample size was computed based on the methodology described in the preceding publication [23]. Such examination was completed from February to July 2025. Of 5561 subjects who were invited and attended the medical centers, we eventually only included subjects with no missing data in the questionnaires and high-quality spirometry graded A or B (Figure 1) [24]. The epidemiological portrait of COPD and occupational profile in the current study were reported elsewhere [23].

### 2.2. Questionnaire

We compiled our questionnaire from a set of international, validated tools created and tested previously in a large number of epidemiological studies. Participating subjects were asked to fill out the questionnaire either in Russian or Kazakh, depending on their personal preference (Table S1). The overall skeleton of the tool was developed and tested before [15], but we now extended the occupational part of that questionnaire. We collected some basic demographic information, including date of birth, sex, place of residence, highest attained education, and even estimated family income per person. This demographic section also included full occupational tenure, and we only considered posts for at least one year.

Because the general population may not be fully aware of specific exposures in the workplace and because recall may affect the accuracy of exposure classification, we decided to apply a job-exposure matrix (JEM) to convert positions held during lifetime into specific exposures. Airborne chemical job-exposure matrix (ACE JEM) was chosen because it offered a very detailed [25] assessment of employment, producing binary variables of exposure to vapor, gas, dust, biodust, mineral dust, fume, diesel, fiber, mist, asbestos and metal. Positions held for a year or more were linked to the Standard Occupational Classification 2000 codes. A composite exposure to VGDF variable was also created. In this study, we only analyzed airborne occupational exposures, because the focus of our analysis was the respiratory system.

The section on smoking status consisted of cigarette smoking, waterpipe smoking and electronic cigarette use questions: “Have you ever smoked more than 100 cigarettes in your lifetime?”, “Do you continue smoking currently?”, “Do you smoke daily?”, “How many cigarettes a day do you smoke?”, and “What is your overall smoking history in years?” These were the questions to ascertain smoking status. Subjects could thus be classified as (a) never-smoker; (b) ex-smoker; and (c) current occasional or daily cigarette smoker. A minimum abstinence of six months was required to classify a subject as an ex-smoker. In ex-smokers, we also asked them to recall the overall smoking duration in years, as well as the time since quitting. Those who quit were asked about the interventions they had chosen to do so, including self-support and their own desire, medical advice and a very brief 1-min intervention, or some aided cessation, including the use of cytisine, nicotine replacement therapy, varenicline or herbal supplements. The question on electronic devices used for smoking or vaping included all possible electronic options, including vapes, nicotine-containing or non-nicotine devices. Therefore, the term “electronic cigarette” in the context of this study assumed both vapes and all forms of electronic cigarettes.

Exposure to environmental tobacco smoke (secondhand smoke) at home or in the workplace was also considered, and one question was included for that. Biochemical verification of smoking status was not planned in this study. Finally, in addition to occupational exposures, cigarette, waterpipe smoking, electronic cigarette use and SHS, we also assessed biomass use for heating and cooking.

For respiratory symptoms classification, we chose the validated CAT questionnaire, which consisted of eight items (scores from 0 to 5 in each question, overall score 0–40). The tool allowed us to distinguish from “fewer symptoms” to “more symptoms” as well as to report the score on a continuous scale indicative of the total symptom burden in a patient. In addition to the overall score, we also separately analyzed questions 1 (cough severity), 2 (sputum production) and 4 (shortness of breath) and reported them in the univariate comparisons.

### 2.3. Spirometry

We followed the technical and interpretative protocols of the European Respiratory Society to perform and interpret spirometry. Reproducible forced expiratory spirometry was accomplished using a calibrated spirometer ‘MAS-2PC’ (Belintelmed LLC, Minsk, Belarus), and reference values were computed with the GLI-2012 reference values set. Only quality A or B spirometry reporting forced expiratory volume in 1 s (FEV1) or forced vital capacity (FVC) [22] was included in the analysis. We reported z-scores (the number of standard deviations from the predicted values) for FEV1, FVC and FEV1/FVC, and additionally selected patients with FEV1/FVC below the lower limit of normal (LLN) for bronchodilation testing. We also measured other volumes and flows, but only FEV1, FVC and FEV1/FVC were analyzed in the current presentation.

### 2.4. Statistical Analysis

In the normality tests, most continuous variables in the dataset were found to be non-normally distributed; therefore, we only applied non-parametric tests for univariate comparisons. Continuous variable distributions are presented as medians with the corresponding interquartile ranges (IQR), whereas categorical data are shown as percentages of the entire group. Continuous variables in the univariate comparisons between two main groups of interest were compared with the Mann–Whitney U test. Binary variables were tested using χ2 tests from contingency tables. Two major groups compared in this analysis were continuing smokers and former smokers. We assume and present throughout that former smokers were those who stopped smoking at some time in the past. Therefore, former smokers were treated equally as quitters.

Basic demographics, exposures of interest and lung function indices, including FEV1, FVC and FEV1/FVC, were compared between continuing smokers and former smokers. In addition to actual FEV1, FVC and FEV1/FVC, we also derived a binary variable of FEV1/FVC below LLN (−1.64 z-scores). We also compared the overall CAT score and selected questions from the CAT between two groups of interest, including question 1 (CAT1), question 2 (CAT2) and question 4 (CAT4).

Variables significantly associated with the probability of being a quitter from such univariate comparisons with *p* < 0.05, in addition to sex, income, highest attained education and even city of residence, were then tested in crude logistic regression models with the same outcome of being a quitter. BMI was first tested as a continuous variable, but we then found its better predictive potential when converted to a binary variable. ROC analysis identified the highest (sensitivity + specificity) for a BMI value more than 25; therefore, BMI more than 25 kg/m^2^ was then used in all the subsequent models. Adjusted model 1 included the variable of interest with age, sex, income, highest attained education, city of residence and BMI, whereas Model 2 was a fully adjusted model for all included variables. Effect measures in our models, both crude and adjusted, were odds ratios (OR) with the corresponding 95% confidence intervals (CI) for each predictor. Additionally, we also tested interactions between predictors. In all tests, *p*-values below 0.05 were considered significant, and NCSS2025 (Utah, USA) was used as statistical software for all tests.

## 3. Results

Overall, we collected data for 5058 participants, but for the current analysis we excluded all never-smokers with the purpose of retaining those with ever-smoking status and those with unacceptable spirometry quality (Figure 1) This cohort (N total 1769) then consisted of current (63%) and former smokers, who quit sometime in the past (37%). Their demographic, smoking, as well as occupational and environmental exposure data are presented in Table 1. Current smokers smoked a median of 10 (IQR 7;20) cigarettes a day for a median of 20 (IQR 8;30) years. Those who quit smoking smoked for 15 (IQR 5;30) years before they ceased smoking, and at the time of examination, remained non-smokers for a median of six (IQR 2;14) years. In addition to cigarette smoking, we found co-exposure to waterpipe smoking (8%) and even concurrent electronic cigarette use (8%). Table 1 also shows that fossil fuel for heating or cooking was used by a very small number of participants (5% or less). The most prevalent exposure was lifetime occupational exposure to VGDF, identified in two-thirds of the cohort. Compared to those who continue to smoke, smoking quitters were older and had a greater BMI (Table 1). In addition, this group was less co-exposed to waterpipe, electronic cigarettes and even occupational exposures. However, the educational background of continuing smokers and quitters did not differ.

Among 663 subjects who stopped smoking and remained non-smokers at the time of this study, 478 (72%) claimed their own desire and successful unaided cessation; 20 (3%) stated they received medical advice and a very brief 1-min intervention; and one patient used cytisine as a pharmacological aid. None of the successful quitters approached behavioral therapy, used nicotine replacement therapy, herbal supplements or varenicline.

**Figure 2:**
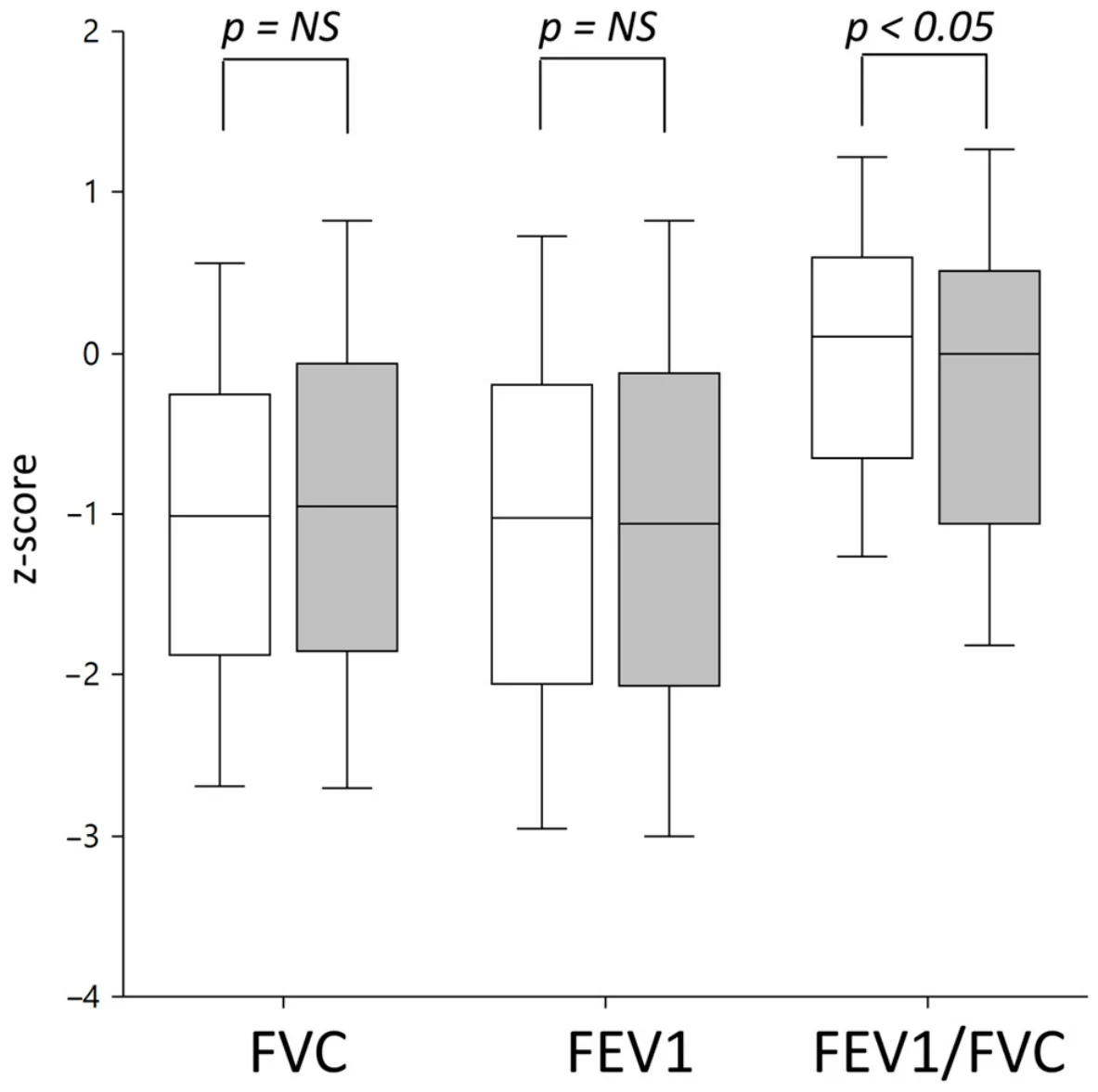
Spirometric indices of quitters with VGDF exposure (gray boxes) and all other subjects (white boxes). Boxes are interquartile ranges; whiskers are 0.5 interquartile ranges. NS—non-significant.

Eleven percent of people in this study were found to have an obstructive ventilation defect (FEV_1_/FVC below LLN), significantly more prevalent in quitters. Therefore, with significantly shorter smoking duration before they stopped compared to the years of smoking in those who continued to smoke, the former had worse lung function and more obstructive subjects. However, when median z-scores of FEV_1_ and FVC were compared between quitters and continuing cigarette smokers, no differences were identified. Similarly, median FEV_1_/FVC z-scores in these two groups did not differ and averaged zero (median value equaled predicted). Furthermore, in quitters with exposure to VGDF (N = 421), FEV_1_/FVC z-score was significantly lower when compared to continuing smokers and quitters with no occupational exposures (median 0 vs. 0.11, *p* < 0.05), but FEV_1_ and FVC z-scores alone were comparable between these two groups (Figure 2). Finally, a group of smoking quitters with occupational exposures had the greatest fraction of obstructive subjects (FEV_1_/FVC below LLN) of all groups (N = 73, 17%) compared to the remaining participants (N = 134, 11%).

**Figure 3:**
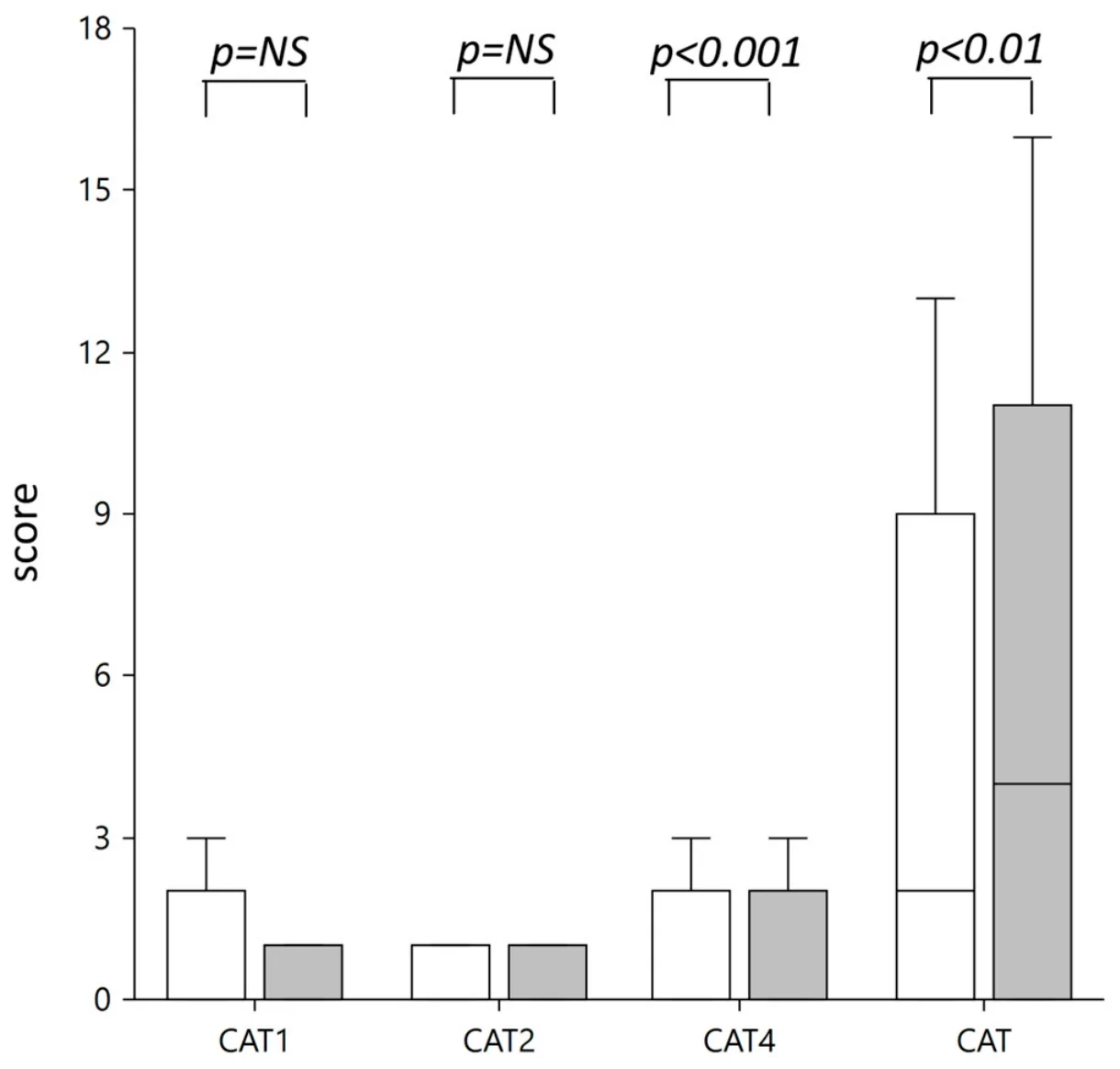
Total CAT and separate three questions of CAT (cough, sputum and dyspnea) scores in quitters (gray boxes) and continuing smokers (white boxes). Boxes are interquartile ranges; whiskers are 0.5 interquartile ranges. NS—non-significant.

In the univariate comparisons of the overall CAT score between continuing smokers and those who quit, we found significantly more symptoms in quitters (median CAT 4 vs. 2) (Figure 3). When three questions of interest were analyzed separately, including CAT1 (cough), CAT2 (sputum) and CAT4 (shortness of breath), the first two were similar in these two groups, but shortness of breath score was highly significantly greater in quitters. This apparently means that, among symptoms, not cough or sputum, but dyspnea was associated with having quit smoking.

Our multivariate models of selected predictors of the probability to be a quitter included age, sex, BMI, income, education level, city of residence, waterpipe smoking, electronic cigarette use, exposure to SHS, lifetime exposure to VGDF, FEV_1_/FVC below LLN and CAT score. Because total CAT and CAT4 (dyspnea) were highly correlated (r = 0.85), we only used overall CAT for all subsequent analyses (Figure 3). In such adjusted models, neither lung function nor symptoms could predict quitting smoking. Furthermore, we also tested FEV_1_, FVC and FEV_1_/FVC z-scores as alternative variables to the binary variable FEV_1_/FVC below LLN, but again found no association with the probability of quitting smoking. In addition to respiratory symptoms and lung function, sex, the city of residence, income, highest attained education and waterpipe smoking showed no association with the outcome. In contrast, age, BMI, lifetime exposure to VGDF, exposure to SHS and even electronic cigarette use were significantly associated with quitting smoking in the adjusted model (Table 2). Thus, SHS increased the odds of continuing smoking, as did occupational exposure to VGDF and the use of electronic cigarettes. On the contrary, advancing age and greater BMI were associated with quitting.

We also identified an interaction between age and FEV_1_/FVC below LLN, as well as between age and BMI. Therefore, we stratified the analyses into a group of subjects with intact lung function and, alternatively, subjects with abnormal lung function. These models indicated that age was significantly associated with quitting only in subjects with normal lung function. Whenever an obstructive defect was present, age and the use of electronic cigarettes were no longer associated with the probability of quitting.

## 4. Discussion

In this population-based study of smokers in five major parts of Kazakhstan, we tested the hypothesis that more advanced respiratory symptoms and worsening lung function, when no clear clinical condition has been formed yet, could be associated with ex-smoking. Therefore, in those who had already stopped smoking, we expected to see subjects with worse respiratory symptoms and poorer lung function. However, our study of almost 2000 subjects older than 18 years (median age 48 years) demonstrated that these had very poor or no association with the number of quitters. Although those who quit had lower FEV1/FVC and their respiratory symptoms score was higher, these two predictors failed to explain the number of former smokers in the multivariate models. Exposure to SHS, use of electronic cigarettes and occupational exposures to VGDF were associated with continuing smoking.

The current study was initiated because most preceding reports yielded discrepant observations. While it is believed that spirometry and feedback on its results, with or without medical advice on respiratory symptoms, increase the likelihood of attempts to quit and those attempts may be successful [10,11,17], some other reports failed to confirm such association, and respiratory symptoms along with lung age may not clearly translate into quit attempts [26,27]. Mechanisms explaining the direction of such association, if any, are hard to find. Quitting smoking is a complex behavioral process progressing through pre-contemplation, contemplation, and further to action, associated with a lot of perils, including immediate social support, smoking policy in the workplace, cigarette affordability and even success stories from peers. Therefore, even if respiratory symptoms are bothering and feedback on spirometry is professional, the probability of quit attempt is very hard to predict. Some studies emphasize that social factors and environmental support were more powerful than symptoms [26]. Therefore, when communicating with respiratory patients, a medical professional should keep in mind that arguments obvious from a medical point of view, may not appeal to a given patient. Although we did not directly assess the association of symptoms and spirometry results with quitting in the past, but only with the number of ex-smokers at the time of examination, our findings may still help plan further prospective studies to test the association of interest.

Individual respiratory health may only be one of the considerations for a smoker to undertake a quitting attempt. In chronic respiratory patients, such as COPD, quitting smoking is essential and determines the future prognosis of natural history, symptom progression, lung function deterioration and even response to treatment [28,29,30]. But in patients with no respiratory disease yet, counseling may face resistance because a subject will not associate his or her morning cough, some sputum and some early shortness of breath with smoking, also given that there is a poor correlation between symptoms and lung function [31]. When a middle-aged subject has not been diagnosed with COPD, respiratory symptoms are mild and lung function impairment is at a very initial stage; such a patient is likely to consider some other reasons to quit rather than respiratory health.

This study uncovered some unexpectedly low use of pharmacological aids and behavioral cessation interventions for smoking cessation. This local context contrasts with the international experience on the use of pharmacological interventions, including varenicline, cytisine and nicotine-replacement therapy, in addition to behavioral support in cessation. We could not identify preceding studies specifically reporting the structure of aided smoking cessation in Kazakhstan published in the biomedical literature, and our novel findings may be indicative of either poor population awareness or poor affordability of these remedies. Further research should focus on the explanatory aspects of such low popularity of pharmacological aids to stop smoking from both safety, efficacy and economic perspectives.

State tobacco control policy is the most powerful strategy to reduce tobacco consumption, also through an increase in quitting rates [32]. Strict governmental tobacco control policy will affect smoking prevalence and increase quit rates, and countries with consistent tobacco control policy have achieved a significant reduction in smoking prevalence, mostly in men. One such control measure is the occupational smoking policy. Smoking bans and restrictions in the workplace may predict more quitting attempts [6,7,33,34,35], including successful ones with long abstinence, than individual conversations with patients, assessing their respiratory symptoms and spirometric lung age, but their comparative analysis has yet to be done. A smoking ban in the workplace is a very cheap but efficient public health intervention, and given that smoking cessation with pharmacotherapy for an individual is costly and very high rates of relapse are reported when no tobacco control policy is reinforced, workplace tobacco control policies should be prioritized.

We found that the use of electronic cigarettes, occupational exposures to VGDF and secondhand smoke were independently associated with greater odds of continuing smoking. These findings have clear public health implications and are supported by earlier larger studies elsewhere [36]. Thus, exposure to SHS increased the odds of smoking initiation in never-smokers [37] and was usually highly prevalent in current smokers, hampering attempts to make smoking not normal and helping smokers stop. We also identified occupational exposures to VGDF to have an inverse association with being an ex-smoker. These findings guide further efforts to identify the least susceptible to contemplation over smoking cessation, including smoking working men exposed to SHS, but a workplace smoking ban can help reduce smoking prevalence and improve lung function in such groups [6,7], given that population attributable fraction from occupational exposures in COPD is high [38].

There are several limitations in our analysis. First, we only relied on self-reported smoking status, when exposure misclassification was likely. In this population-based design in remote cities, biochemical verification, even with exhaled CO, was hard to organize. Secondly, the cross-sectional design did not allow us to prospectively evaluate smoking status, and we could not confirm temporality. Thus, respiratory symptoms and lung function in a quitter may have improved sometime after cessation, thus biasing the association of symptoms and lung function with being an ex-smoker at the time of examination, explaining why the association of symptoms and lung function with quit rates was not significant. Thirdly, we could not take into account health status other than respiratory in this analysis, knowing that cardiovascular health, diagnosed coronary artery disease (CAD), or even intervention for CAD could also have affected quit rates and intention to quit rates. Finally, because all exposure data were collected using questionnaires, recall bias cannot be ruled out.

We emphasize clear implications for public health practice generated in our study. When occupational exposures are combined with exposure to SHS in current smokers, such subjects appear to be the least likely to quit. Thus, targeted interventions to promote quitting attempts for early COPD prevention should be prioritized in relatively healthy middle-aged men in hazardous workplaces. Furthermore, respiratory symptoms in cigarette smokers, as well as lung function impairment at early stages when COPD is not yet established, are not sufficient to undertake quitting attempts; therefore, health messages about benefits of cessation in primary prevention may not be as efficient as medical professionals would expect. Other directions to promote transfer from contemplation to action and cessation, including economic losses, social deprivation, poor role modeling for children and others, should also be considered in current smokers.

## 5. Conclusions

Respiratory symptoms and lung function alone were found not to have an association with being an ex-smoker. Co-exposure to secondhand smoke, occupational exposures to VGDF and electronic cigarette use were associated with continuing smoking. Interventions to promote cessation in current smokers for early COPD prevention and non-smokers’ health protection may additionally consider not only the health-related benefits of cessation, but also take into account other ways of motivation; their effect is to be tested in future cohort observations.

## Figures and Tables

**Table 1:** Demographic, lifestyle and exposure data of study ever-smoking participants.

Variable	Overall	Current Smokers	Former Smokers
N (%)	1769 (100)	1106 (63)	663 (37)
Age, years *	48 (33; 60)	45 (31; 56)	52 (37; 64)
Men, N (%)	1480 (84)	934 (84)	546 (83)
Height, cm *	173 (168; 178)	174 (168; 178)	172 (167; 178)
Weight, kg *	77 (67; 89)	76 (65; 88)	78 (70; 90)
BMI, kg/m^2^ *	25.9 (22.7; 29.3)	25.3 (22.2; 28.9)	26.6 (23.5; 29.7)
Highest attained education			
Secondary school	28 (2)	22 (2)	6 (1)
High school	497 (28)	314 (30)	183 (28)
College	699 (40)	443 (40)	256 (39)
University degree	535 (30)	323 (30)	212 (32)
Academic degree	10 (0)	4 (1)	6 (0)
Waterpipe smoking, N (%) *	148 (8)	111 (10)	37 (6)
Electronic cigarette use or vaping, N (%) *	135 (8)	106 (10)	29 (4)
Exposure to secondhand smoke, N (%) *	883 (50)	672 (61)	211 (32)
Use of fossil fuel for cooking, N (%)	53 (3)	31 (3)	22 (3)
Use of fossil fuel for heating, N (%)	80 (5)	46 (4)	34 (5)
Lifetime exposure to VGDF, N (%) *	1186 (67)	765 (69)	421 (63)
FEV_1_, z-score	−1.04 (−2.06; −0.17)	−1.05 (−2.05; −0.23)	−1.02 (−2.08; −0.13)
FVC, z-score	−1.00 (−1.87; −0.23)	−0.99 (−1.88; −0.23)	−1.00 (−1.87; −0.22)
FEV_1_/FVC, z-score	0 (−0.75; 0.59)	0 (−0.71; 0.55)	0 (−0.87; 0.61)
FEV_1_/FVC below LLN, N (%) *	207 (11)	114 (10)	93 (14)

Note: *—*p* < 0.05 in either Mann–Whitney U test for continuous variables or χ^2^ test for categorical variables; VGDF—vapors, gases, dusts and fumes; FEV_1_—forced expiratory flow in one second; FVC—forced vital capacity; LLN—lower limit of normal.

**Table 2:** Crude and adjusted models of selected predictors of being a smoking quitter.

Variable	Crude OR	Model 1	Model 2
Age	1.02 (1.02; 1.03)	1.02 (1.02; 1.03)	1.02 (1.01; 1.03)
Male sex	0.86 (0.66; 1.11)	0.96 (0.74; 1.26)	1.05 (0.79; 1.39)
City (Karaganda, Ust-Kamenogorsk and Aktobe vs. Shymkent and Kostanay)	1.32 (1.09; 1.60)	1.19 (0.96; 1.46)	1.17 (0.94; 1.47)
Higher income	1.02 (0.78; 1.34)	1.11 (0.84; 1.47)	1.07 (0.79; 1.43)
College, university or higher level of education	1.09 (0.89; 1.35)	1.08 (0.86; 1.36)	1.16 (0.91; 1.47)
BMI more than 25 kg/m^2^	1.55 (1.27; 1.89)	1.37 (1.12; 1.68)	1.36 (1.10; 1.68)
Lifetime exposure to VGDF	0.78 (0.63; 0.95)	0.71 (0.57; 0.88)	0.77 (0.62; 0.97)
Electronic cigarette use	0.43 (0.28; 0.66)	0.61 (0.39; 0.96)	0.58 (0.34; 0.99)
Waterpipe smoking	0.53 (0.36; 0.78)	0.76 (0.51; 1.15)	1.10 (0.67; 1.82)
Exposure to secondhand smoke	0.30 (0.25; 0.37)	0.33 (0.27; 0.41)	0.34 (0.27; 0.42)
CAT score	1.02 (1.01; 1.03)	0.99 (0.98; 1.00)	0.99 (0.97; 1.00)
FEV_1_/FVC below LLN	1.42 (1.06; 1.90)	1.01 (0.73; 1.40)	1.07 (0.74; 1.50)

Note: Data shown as odds ratios with the corresponding 95% confidence intervals; FEV_1_—forced expiratory flow in one second; FVC—forced vital capacity; LLN—lower limit of normality; VGDF—vapor, gas, dust and fume; BMI—body mass index; CAT—COPD Assessment Test; Model 1 adjusted for age, sex, income, education, city and BMI; Model 2 adjusted for all listed variables.

## Data Availability

The data presented in this study are available on request from the corresponding author due to the considerable size of the data files.

## Supplementary Materials

The following supporting information can be downloaded at https://www.mdpi.com/article/10.3390/jcm15197502/s1, Table S1: Questionnaire for a study participant.

## Abbreviations

The following abbreviations are used in this manuscript:

ACE JEM	airborne chemical job-exposure matrix
BMI	Body mass index
CAT	COPD Assessment Test
CI	confidence interval
COPD	chronic obstructive pulmonary disease
FEV_1_	forced expiratory volume in one second
FVC	forced vital capacity
IQR	interquartile range
LLN	lower limit of normality
OR	odds ratio
SHS	secondhand smoke
VGDF	vapor, gas, dust and fume
