# Supplementary material for: Lung Function and Respiratory Symptoms as Factors Associated with Smoking Cessation Status in a Population-Based Study
Source: J Clin Med. 2026 Sep 26;15(19):7502. doi: 10.3390/jcm15197502 (PMC13666024; doi:10.3390/jcm15197502)
Supplement: Supplementary file 1 [file jcm-15-07502-s001.zip › jcm-4517312-supplementary.pdf]

Table S1. Questionnaire for a study participant

1. Last name, first name, acronym ____
2. Mobile phone ______________________________
3. Date of birth (DD/MM/YYYY) _________________
4. Sex at birth: ________________________________
5. Permanent address:____
6. Ethnicity in passport: ____________________
7. Please list all positions you help in your lifetime for more than a year starting from the most recent:

Position	Organization (a plant, company name)	Tenure (years)	SCO

8. Please choose the highest attained education:
   □ Secondary school
   □ High school
   □ College
   □ University
   □ Academic degree (PhD, doctor of science)
9. What is your approximate family income a month?
   □ below 100 thousand tenge
   □ 101-500 thousand tenge
   □ 501 thousand tenge or more
10. Have you even smoked more than 100 cigarettes in your life? □ No (tick and jump to question №17) □ yes
11. Do you continue smoking at present?
    □ No (tick and jump to question №17)
    □ Yes (tick and jump to question №14)
12. What was you smoking duration if you quit? ________ 12a. What is your full abstinence duration? _______
13. How did you stop smoking? □by myself □doctor's advice □behavioral therapy
    □nicotine replacement therapy □Tabex □Champix □ Herbal supplements
14. Do you smoke cigarettes daily? □ No (tick and jump to question №17)
    □ Yes
15. How many cigarettes a day on average do you smoke? _______
16. What is your overall smoking duration? _________
17. Are you exposed to smoke from others at home or in the workplace? □ No □ Yes
18. Do you smoke waterpipe? □ No (tick and jump to question №20)
    □ Yes
19. How many times month do you smoke waterpipe? ______________ times a month.
20. Do you use any electronic devices for heating, smoking or vaping? □ No □ Yes
21. In your house of apartment, do you use biomass for heating (other than natural gas)? □ No □ Yes
22. In your house of apartment, do you use biomass for cooking (other than natural gas)?□ No □ Yes
23. CAT. In each question, please choose from 0 to 5:

1	I never cough	0 1 2 3 4 5	I cough all the time
2	I have no phlegm (mucus) in my chest at all	0 1 2 3 4 5	My chest is completely full of phlegm (mucus)
3	My chest does not feel tight at all	0 1 2 3 4 5	My chest feels very tight
4	When I walk up a hill or one flight of stairs I am not breathless	0 1 2 3 4 5	When I walk up a hill or one flight of stairs I am very breathless
5	I am not limited doing any activities at home	0 1 2 3 4 5	I am very limited doing activities at home
6	I am confident leaving my home despite my lung condition	0 1 2 3 4 5	I am not at all confident leaving my home because of my lung condition

7	I sleep soundly	0 1 2 3 4 5	I don’t sleep soundly because of my lung condition
8	I have lots of energy	0 1 2 3 4 5	I have no energy at all
